# Spatial–temporal analysis and spatial drivers of hepatitis-related deaths in 183 countries, 2000–2019

**DOI:** 10.1038/s41598-023-45672-5

**Published:** 2023-11-13

**Authors:** Jie Li, Zejia Xu, Hong Zhu

**Affiliations:** 1 School of Geographical Sciences and Remote Sensing, Guangzhou University, Guangzhou, 510006 China; 2 Key Laboratory of Philosophy and Social Sciences in Guangdong Province of Maritime Silk Road of Guangzhou University (GD22TWCXGC15), Guangzhou, 510006 China; 3 School of Geography and Planning, Ningxia University, Yinchuan, 750021 China

**Keywords:** Viral hepatitis, Public health, Risk factors

## Abstract

Hepatitis is the seventh leading cause of mortality worldwide and is the only communicable disease where mortality is increasing, yet the long-term spatial–temporal variation at global scale and its possible causes, i.e., drivers, remain unknown. Firstly, this study employed the measure of spatial autocorrelation, Moran’s I, and the measure of local spatial cluster, Getis-Ord G_i_*, to characterize the spatial variation of mortality due to hepatitis in 183 countries globally for years 2000, 2010, 2015 and 2019. Then, a novel spatial statistical method, named the Geographical Detector, was utilized to investigate eight possible influencing factors, i.e., risk factors, of the spatial–temporal variation of mortality due to hepatitis. The results showed significant disparities of hepatitis-related mortality rates among countries. Hot spots, representing locations with higher mortality rates, were consistently observed in Africa, East Asia, and Southeast Asia, while the cold spots, representing locations with lower mortality rates, were predominantly found in Europe and the Americas. Potential spatial drivers of hepatitis mortality, identified by geographical detector, include “health expenditure”, “universal health coverage”, and “per capita income”. However, “hepatitis B immunization” and “total population” were not identified as significant spatial drivers for hepatitis mortality The findings highlighted the critical role of socioeconomic factors in the variations in hepatitis mortality, and pointed out relative importance of increasing health expenditure, per capita income, and improve universal health coverage on alleviating global hepatitis-related mortality.

## Introduction

Viral hepatitis is an infection that causes liver inflammation and damage. It is caused by several hepatitis virus, and poses a substantial global health burden^1,2^. In 2019, hepatitis ranks among the top 20 causes of death globally, accounting for 2.0% of deaths from all causes worldwide^3,4^. Viral hepatitis caused 1.1 million deaths, with approximately 354 million chronic infections and about 3.0 million new infections annually^3,5^. Recent Global Burden of Disease study (GBD) estimates indicate a high morbidity and mortality of viral hepatitis attributable to chronic hepatitis^6^.

Hepatitis remain a non-negligible health threat globally^2^, and prior studies have elucidated morbidity^7–9^, mortality^5,7,10^ and risk factor^8,10,11^ associated with hepatitis. Notably, GBD provides a comprehensive framework for hepatitis research. These studies based on GBD have estimated the global, regional and national burden of viral hepatitis^1,6^, illustrated the development of the burden, and suggested that multiple risk factors contribute to the variation of hepatitis^8,12^. However, these studies considered countries and regions as independent units, overlooking the geographic relationships and spatial patterns of morbidity and mortality of hepatitis. Significant spatial variations exist across countries with different socioeconomic status, with developing countries affected most ^13,14^. Spatial analyses offer insights into spatial patterns, areas experiencing significant changes in hepatitis burden, and the spatial relationships between hepatitis burden and social, economic and environmental factors^15–17^. However, there is a lack of studies investigating long-term and large-scale global variations of mortality due to hepatitis.

Despite the availability of highly efficacious hepatitis vaccines, there has been no substantial decrease in hepatitis-related deaths over the past two decades, underscoring the ongoing public health burden^18^. Unfortunately, hepatitis receives comparatively less attention than other major infectious diseases, despite causing comparable health burdens as HIV, tuberculosis, and malaria^2,7,18,19^.

Furthermore, despite the demonstrated importance of economic burden on viral hepatitis prevention and treatment^11,13^, these GBD studies lack factors directly related to social and economic status^8,10,11^. Thus, the incorporation of socioeconomic factors is essential for a comprehensive understanding of hepatitis-related health outcomes.

This study aims to conduct a long-term and large-scale investigation to identify and comprehend the spatial patterns of hepatitis-related mortality, and reveal the spatial relationships between mortality and socioeconomic risk factors. By utilizing data on hepatitis-related deaths and mortality rates, this study presents variations of hepatitis-related deaths and mortality rates using spatial statistics, and characterized the spatial patterns of hepatitis-related mortality through spatial autocorrelation analysis. Additionally, from the Sustainable Development Goals (SDGs), we collected socioeconomic factors to quantify their impact on hepatitis-related mortality through spatial heterogeneity analysis. All the factors are analyzed using the Geographic Detector, which was used to quantify spatial heterogeneity, in this study. Our research contributes to advancing the understanding of hepatitis-related mortality dynamics and provides crucial evidence for targeted public health interventions.

## Methods

### Data sources

Hepatitis-related deaths encompass causes of death strongly associated with hepatitis, including deaths from acute hepatitis infection, liver cancer, and cirrhosis^19^. These deaths are defined in the International Classification of Diseases 11th Revision (ICD-11) with codes 1E50, 1E51, 2C12.02 and DB93.1^20^. Data on hepatitis-related deaths and mortality were collected from the Global Health Observatory, a database of the World Health Organization (WHO). This database provides summary estimates of deaths and mortality by cause for the years 2000, 2010, 2015 and 2019 across 183 countries^18,21^. To account for confounding effects from the population age structure, the age-standardized mortality rate was employed to reflect hepatitis-related mortality, expressed as a number per 100,000 population. In addition, to highlight regional variations in hepatitis-related deaths, the 183 countries were categorized into ten regions based on geographic location and SDG (Sustainable Development Goals) classification^22^ (Table 2).

Hepatitis-related socioeconomic factors are those directly related to hepatitis or contribute to hepatitis indirectly, involving social economy, sanitation and immunization (Table 1). Hepatitis-related socioeconomic status was collected from SDGs indicators, for the reason that health-related SDGs represent a critical component of health-related socioeconomic status, providing a comprehensive framework encompassing various determinants of health^23^. The factors selected include those related to national health conditions, strategies, and policies^5,24^, and were collected from the Global Health Observatory^25–27^. Furthermore, per capita income data were obtained from the World Bank Country and Lending Groups^28^, categorized into four groups based on the World Bank Atlas method: low-income countries, lower-middle-income countries, upper-middle-income countries and high-income countries^18^. Total population data were collected from World Population Prospects: The 2019 Revision^22^.

**Table 1:** Health-related socioeconomic status and corresponding risk factors category.

Socioeconomic status	Risk factor	Health SDGs
Health services	Per capita health expenditure	SDGs 1.a.2
	per capita government health expenditure	
	Universal health coverage	SDGs 3.8.1
Drinking water and sanitation	Basic sanitation services	SDGs 6.2.1
Prevention and treatment	Hepatitis B incidence	SDGs 3.3.4
	Hepatitis B immunization	SDGs 3.b.1
Population	Total population	–
Economic growth	Per capita income	–

### Spatial–temporal analysis

Spatial statistical methods were employed to reveal the temporal variation and the distribution of hepatitis-related mortality rate of 183 countries from 2000 to 2019. The methods used included standard deviation ellipse (SDE), Global Moran’s I and Getis-Ord *G*_i_*.

The standard deviation ellipse is a spatial statistical method used to assess the concentration of geographic features, revealing the spatial distribution through the center, orientation and concentration of the ellipse^29, 30^. In this study, we applied the SDE to represent the concentration of hepatitis-related mortality rates and compared the SDEs for different years to reveal temporal variation in the distribution of hepatitis-related mortality rates. The formula for the calculation is as follows:

1 $${SDE}_{x}=\sqrt{\frac{\sum_{i=1}^{\mathrm{n}}{({\mathrm{x}}_{i}-\overline{\mathrm{x}})}^{2}}{n}}$$

2 $${SDE}_{y}=\sqrt{\frac{\sum_{i=1}^{\mathrm{n}}{({\mathrm{y}}_{i}-\overline{\mathrm{y}})}^{2}}{n}}$$

where *n* is the total number of countries; ${\mathrm{x}}_{i}$ and ${\mathrm{y}}_{i}$ is the geographic coordinates of country *i*; $\overline{\mathrm{x}}$ and $\overline{\mathrm{y}}$ is the average center of countries.

Moran’s *I* is a widely used method for assessing spatial autocorrelation, which characterizes the spatial distribution of attributes by measuring clustering, dispersion or outliers^15^. In this study, we applied the global Moran’s *I* to determine the global spatial autocorrelation of hepatitis-related mortality rate. The equation for the calculation is as follows:

3 $$I=\frac{n}{{\sum }_{i=1}^{n} {\sum }_{j=1}^{n}{w}_{i,j}}\frac{{\sum }_{i=1}^{n} {\sum }_{j=1}^{n}{W}_{i,j}\left({x}_{i}-\overline{x }\right)\left({x}_{j}-\overline{x }\right)}{{\sum }_{i=1}^{n}{\left({x}_{i}-\overline{x }\right)}^{2}}$$

where *n* is the total number of countries; ${x}_{i}$ and ${x}_{j}$ are hepatitis-related mortality rates of country *i* and *j* (where *i* ≠ *j*); $\overline{\mathrm{x}}$ is the average over all locations of countries; ${w}_{i,j}$ is the spatial weight between country *i* and *j*. Spatial weight quantifies the spatial relationships or connectivity between different countries, and we used a distance-based spatial Weight in this study. The value of Moran’s *I* ranges from -1 to 1; when Moran’s *I* > 0, the mortality is spatially clustered; when Moran’s *I* < 0, the mortality is dispersed; and when Moran’s *I* = 0, the features are randomly distributed. The *Z*-score and *P*-value provide statistical significance on the calculated Moran’s *I* using a 95% confidence level.

Getis-Ord *G*_i_* is a spatial statistic used to describe the local spatial autocorrelation of the given spatial feature^31,32^, here hepatitis-related mortality rate. It identifies whether there is high- or low-value concentration of the hepatitis-related mortality rate for each country. Getis-Ord *G*_i_*, on the other hand, is local spatial autocorrelation index. The equation for calculation is as follows:

4 $${G}_{i}^{*}= \frac{{\sum }_{j=1}^{n}{w}_{i,j}{x}_{j}-\overline{x}{\sum }_{j=1}^{n}{w}_{i,j}}{{\sum }_{j}^{n}{w}_{i,j}{x}_{j}}$$

where ${w}_{i,j}$ is the spatial weight between country *i* and *j*. Hot spots indicate that country *i* is surrounded by countries with high values of mortality, while cold spots indicate that country *i* is surrounded by countries with low values of mortality. Getis-Ord *G*_i_* can further detect these hot spots and cold spots, and divide them into high-significant spots, medium-significant spots and low-significant spots based on statistical significance (*P*-value).

Statistical analyses were perform using the R (v4.3.0, https://www.r-project.org/). Spatial analyses, including the standard deviation ellipse, Global Moran’s *I* and Getis-Ord G_i_*, were implemented in ArcGIS Pro (v2.8, ESRI, Redlands, CA, USA). For world map visualization and spatial analyses, we utilized the Compact Miller projection, chosen for its suitability in displaying global coverage^33^.

### Spatial heterogeneity analysis

To investigate the correlation between hepatitis-related mortality and socioeconomic risk factors, we conducted spatial heterogeneity analysis using the Geographical Detector, a spatial statistic designed to detect spatial stratified heterogeneity of spatial features^34–36^. Geographical Detector does not require a linear hypothesis to reveal the factors behind spatial stratified heterogeneity^34^. We utilized geographical detector to quantify spatial heterogeneity and assess the interaction effects on eight risk factors for hepatitis-related mortality, determining whether risk factors influenced the observed spatial pattern. The equation for the calculation is as follows:

5 $$q=1-\frac{\sum_{h=1}^{L}{N}_{h}{\sigma }_{h}^{2}}{N{\sigma }^{2}}$$

where *N* and ${\sigma }^{2}$ stand for the number of units and the variance of variable in a study area, respectively. *h* = 1, 2, …, *L* is strata of variable. The *q*-statistic ranges from 0 to 1with 0 indicating that the explanatory power of a given factor is not significant, and 1 indicating that the explanatory is perfect. To perform the analysis, we reclassified hepatitis-related mortality rate and eight of the eight risk factors into five levels using the natural break classification method^37^. Per capita income and universal health coverage were used with the levels provided by the data sources^28, 38^. Spatial heterogeneity analysis was performed using Geodetector software (http://geodetector.cn/).

The potential risk factors were first classified into classes/strata according to existing classifications, regulations, expert knowledge, or characteristics of the data. Then, the classes were loaded into Geographic Detector. Thirdly, factor detector in Geographic Detector was used, and the percent of spatial variation in mortality due to hepatitis explainable by spatial variation the risk factors/indicators were quantified. Those that are statistical significant were selected as potential factors that may drive the spatial–temporal pattern of hepatitis-related mortality, i.e., drivers.

## Results

### Spatial–temporal variation in hepatitis-related deaths and mortality

From 2000 to 2019, the global hepatitis-related deaths showed a slight increase from 1.14 million to 1.15 million, while the age-standardized mortality rate experienced significant decline from 23.59 to 16.29(Table 2). Despite this reduction, viral hepatitis deaths remained a major global health challenge.

**Table 2:** Hepatitis-related deaths and mortality rate in the world at (sub) continental level between 2000 and 2019.

Region	Number of deaths ('000)				Age-standardized mortality rate per 100,000			
	2000	2010	2015	2019	2000	2010	2015	2019
Africa	184.03	204.25	207.22	212.00	41.32	34.03	29.47	26.68
Central Asia	9.82	10.48	10.18	9.86	24.66	23.60	20.06	17.42
East Asia	402.37	280.63	274.72	289.51	37.54	33.36	28.81	26.69
Europe	88.65	99.36	93.75	90.67	8.62	7.81	6.81	6.40
North America	45.03	55.97	64.55	67.02	9.12	8.21	8.50	8.02
Oceania	1.87	2.52	3.40	3.59	23.45	20.72	20.58	19.68
South America	25.48	28.35	30.00	31.24	9.87	8.13	7.32	7.05
South Asia	229.84	259.62	256.63	258.91	24.40	18.80	16.33	15.39
Southeast Asia	126.13	138.94	143.94	156.39	32.07	27.88	24.86	23.90
Western Asia	28.47	28.69	31.76	34.42	19.50	15.57	14.01	13.18
World	1141.68	1108.80	1116.15	1153.61	23.59	19.88	17.63	16.29

Hepatitis-related mortality experienced a decreasing trend across all countries globally, while the number of deaths increased consistently. Africa had the highest hepatitis-related mortality, followed by East Asia and Southeast Asia, where significant decreases were observed. Meanwhile, these regions accounted for 57.0% of global hepatitis-related deaths. In contrast, Europe, North America, and South America had the lowest observed hepatitis-related mortality rates, remaining stable or showing a slight decrease.

The geographic distribution of hepatitis-related mortality varied among countries from 2000 to 2019 (Supplementary Table S1 and S2). Egypt, Mongolia, and Cambodia, located in the regions with the highest hepatitis-related mortality rates, had the three highest mortality rates. Notably, the hepatitis-related mortality rate in these countries was significantly higher than that of their neighbors. Conversely, Norway, located in the lowest mortality region, had the lowest hepatitis-related mortality rate. Furthermore, in the Africa region, most countries had hepatitis-related mortality rates significantly higher than the global average. In Europe, North America and South America regions, the hepatitis-related mortality rate in almost all countries was lower than the world average. In the remaining regions, most countries had hepatitis-related mortality rates close to the global average, but with notable differences between countries.

### Global trend and spread in hepatitis-related mortality

**Figure 1:**
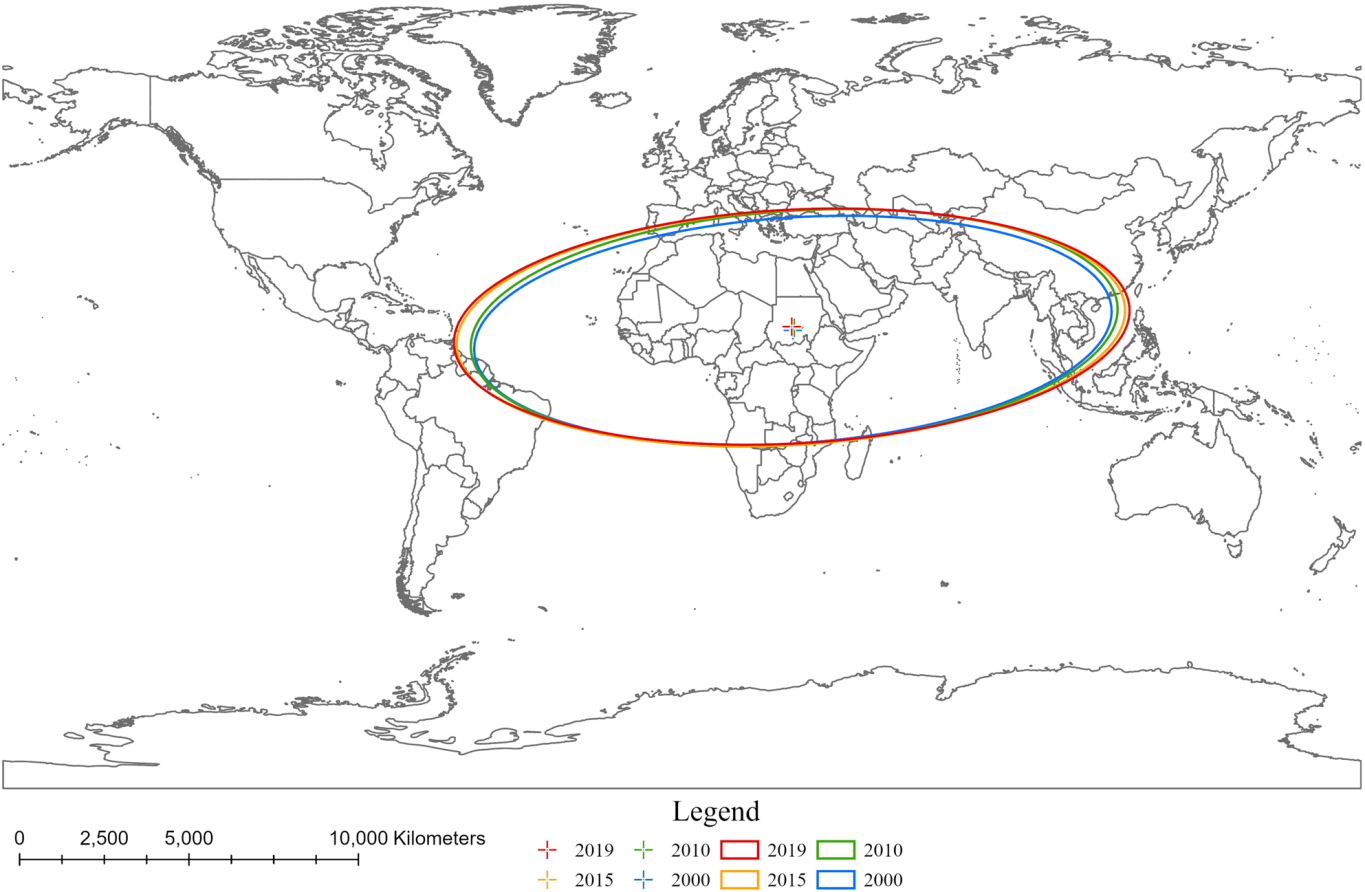
Standard deviation ellipse of hepatitis-related mortality rate between 2000and 2019.

The SDE depicted in Fig. 1 was employed to examine the trend and spread of hepatitis-related mortality rates. Globally, the main axis of hepatitis-related mortality rate was sustained with a southwest-northeast direction, with the long- and short-axis of the SDE mainly covered regions of Africa, West Asia, South Asia and Southeast Asia regions, showing higher hepatitis-related mortality rates. Meanwhile, the SDE presented a gradual increasing trend, particularly in the northwest and eastern directions. This expansion of SDE indicates that a sustainable reduction in the disparities of hepatitis-related mortality rates over time. Given the temporal variations of mortality rates suggests this expansion was driven by substantial reductions in countries with high mortality rates, while countries with low mortality rates remained stable.

**Figure 2:**
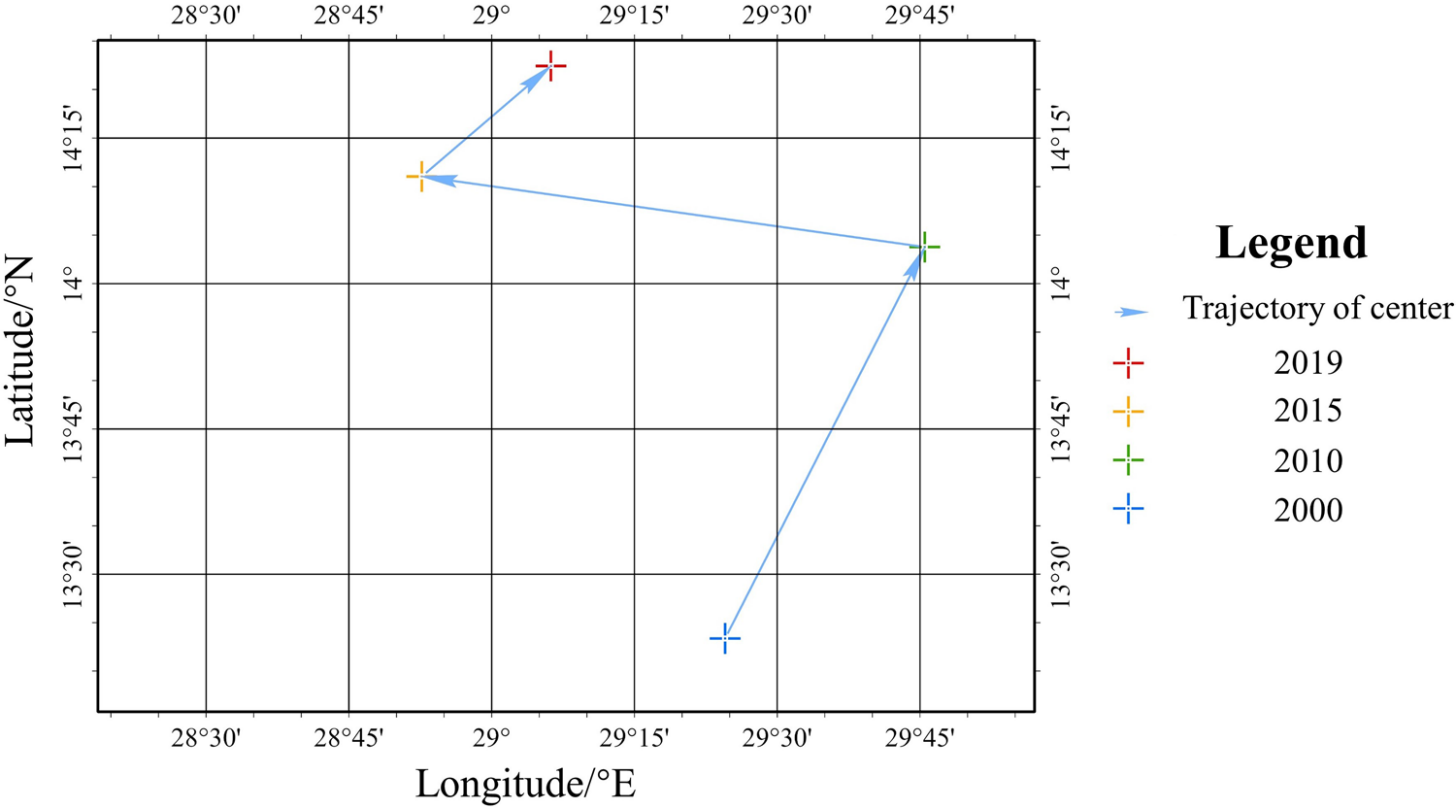
Trajectory of center of gravity of hepatitis-related mortality rate between 2000 and 2019.

Figure 2 depicted the variation in the center of gravity of hepatitis-related mortality rate. From 2000 to 2010, the center of gravity shifted significantly northeastward, followed by a notable northwestward shift from 2010 to 2015, and a slight northeastward movement from 2015 to 2019. Generally, variations in the center of gravity of the hepatitis-related mortality rates presented a distinct trend in the longitudinal direction, with the center of gravity gradually shifting northward. This trend indicates that the substantial reductions in mortality rates primarily occurred the countries located in the southern part of the ellipse.

### Spatial patterns of hepatitis-related mortality

From 2000 to 2019, Moran’s *I* consistently showed positive values (Table 3), indicating persistent spatial clustering of hepatitis-related mortality rate. Based on the high *Z*-score, the spatial clustering of hepatitis-related mortality rate was statistically significant for high degree (*Z*-score greater than 1.96 is statistically significant). Despite decreasing values of Moran’s *I* and *Z*-score, the clustering pattern remained unchanged, indicating stable spatial distribution of hepatitis-related mortality rates. This suggests that neighboring countries tended to have similar values of hepatitis-related mortality rate, with high-mortality countries clustering with other high-mortality countries and vice versa for low-mortality countries.

**Table 3:** Global Moran’s *I* of hepatitis-related mortality rate globally.

Year	Moran's *I*	*Z*-score	*P*-value
2000	0.477	19.014	0.000
2010	0.401	16.190	0.000
2015	0.356	14.516	0.000
2019	0.351	14.289	0.000

**Figure 3:**
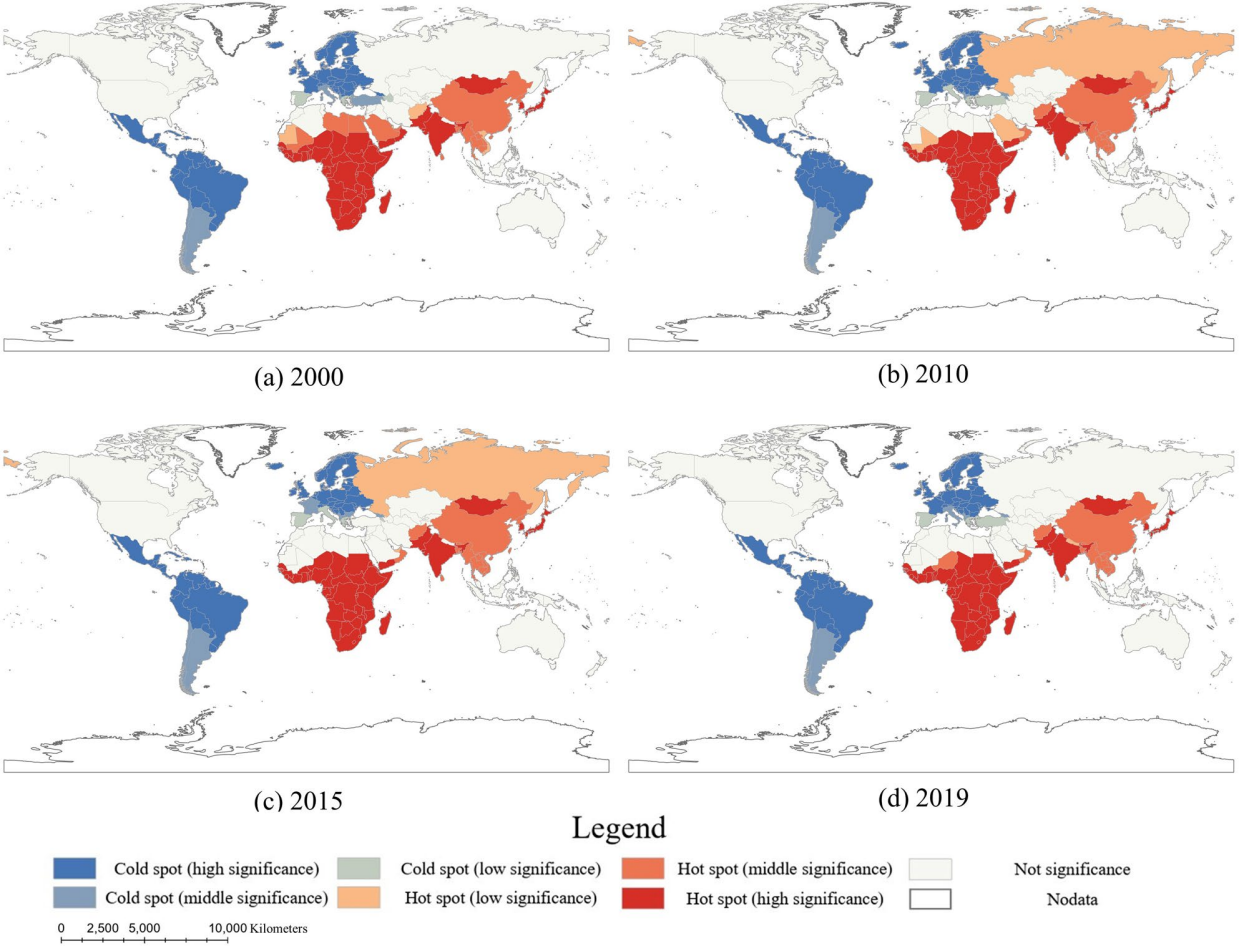
Getis-Ord *G*_i_* of hepatitis-related mortality rate between 2000 and 2019.

Countries with significant clustering of high and low values of hepatitis-related mortality rates were identified using the Getis-Ord *G*_i_* statistic (Fig. 3A–D). The spatial clustering pattern of hepatitis-related mortality rates remained stable over the past two decades. In 2019, high-value clustering (hot spots) was predominantly observed in Africa, South Asia, Southeast Asia and East Asia regions. Hot spots with high significance were identified in Sub-Saharan Africa, South Asia and East Asia. In addition, Yemen, Mongolia, South Korea and Japan showed higher significance than neighboring countries. Interestingly, the hot spots with low significance occurred in Russia in 2010 and 2015. In contrast, the low-value clustering (cold spots) was concentrated in Europe, North America and South America regions, particularly in Latin America and the Caribbean. Cold spots in Europe and South America exhibited high significance, with a few exceptions (Fig. 3D). These results highlighted that high hepatitis-related mortality rates were predominantly concentrated in developing countries, particularly in sub-Saharan Africa, while mortality rates were significantly lower in developed countries.

### Socioeconomic factors in the distribution of hepatitis-related mortality

The geographical detector was employed to quantify the associations of the eight socioeconomic factors on hepatitis-related mortality rate in 2019 (Table 4). Among eight factors, six factors showed significantly influences (*p* < 0.05) on hepatitis-related mortality rate, though their extents varied. Generally, hepatitis-related mortality rate is prominently associated with health expenditure, per capita income and universal health coverage. The leading factor was per capita government health expenditure (*q* = 0.5752), closely followed by per capita health expenditure (*q* = 0.5694), indicating that health expenditure was the crucial factor for hepatitis-related mortality. In addition, per capita income, universal health coverage, and basic sanitation services also demonstrated considerable associations with hepatitis-related mortality rate. While the *q*-statistics for some of the significant factors were relatively low, their effects on hepatitis-related mortality remained non-negligible. In contrast, total population and hepatitis B immunization did not show significant associations with hepatitis-related mortality rate. The total population had only a minor influence on hepatitis-related mortality rate, with a *q*-statistic of 0.005. Notably, despite the well-documented efficacy of the hepatitis B vaccine in reducing hepatitis B incidence, our analysis found no significant association of hepatitis B immunization with hepatitis-related mortality rate.

**Table 4:** Stratified spatial heterogeneity of countries as quantified by *q*-statistics calculated by the geographical detector.

Socioeconomic status	Risk factor	Q-statistic	*P*-value
Health services	Per capita health expenditure	0.575	0.000
	Per capita government health expenditure	0.569	0.000
	Universal health coverage	0.562	0.000
Drinking water and sanitation	Basic sanitation services	0.508	0.000
Prevention and treatment	Hepatitis B incidence	0.326	0.000
	Hepatitis B immunization	0.102	0.104
Population	Total population	0.005	0.998
Economic growth	Per capita income	0.521	0.000

## Discussion

This study presented a comprehensive characteristic of hepatitis-related deaths and mortality in 183 countries from 2000 to 2019. In 2019, the global hepatitis-related deaths reached 1.15 million, showing a slight increase. Despite the consistent absolute number of hepatitis-related deaths at approximately 1.10 million per year, hepatitis-related mortality rate witnessed a steady decline of 44.8%. Notably, the most substantial reduction in hepatitis-related mortality occurred in Africa, East Asia and South Asia regions. Furthermore, this study investigates the correlations of socioeconomic factors, such as health expenditure, per capita income, and universal health coverage.

Estimates of hepatitis-related deaths underscore the ongoing global health burden by viral hepatitis. This study revealed diverse trends in hepatitis-related deaths across regions. With the exception of East Asia, the remaining regions have witnessed a steady or stable increase in hepatitis-related death numbers in recent decades. In contrast, hepatitis-related mortality rates have shown a significant downward trend across regions, particularly in Africa, East Asia and South Asia, with declines of 54.9%, 40.6% and 58.6%, respectively. Notably, East Asia has experienced significant reductions in both death numbers and mortality rates, with a decrease of 0.11 million and 6.67 per 100,000 population, respectively. This reduction in East Asia, exemplified by China, is attributed to enhanced preventive interventions and efforts to address health inequities within the country^39,40^. Consequently, effective interventions should focus on improving coverage of health care services for hepatitis^5,39^.

This study revealed significant clustering of hepatitis-related mortality rates in countries located in Africa and Asia. While hepatitis-related mortality varied across countries, countries with high-value clustering presented significant concentration of distributions. The regions most affected by this high-value clustering include Sub-Saharan Africa, South Asia, Southeast Asia, and East Asia, primarily comprising developing countries. Sub-Saharan Africa and South Asia were the most prominent in the high-value mortality clustering, with all countries showing high significant clustering. In Southeast Asia and East Asia, excluding countries in the Malay Archipelago, the remaining countries presented varying degrees of high-value mortality clustering. Notably, Mongolia and Cambodia experienced exceptionally high mortality rates compared to their neighboring countries. Furthermore, despite relatively low hepatitis mortality rates in Russia, the country underwent a fluctuating pattern of increase and subsequent decrease, leading to localized hot spots. Given the vast size and proximity to high mortality countries, Russia faces an increasing risk of hepatitis transmission and mortality. These findings highlight the broader scope of countries with high hepatitis mortality and underscore the need for regional, collaborative efforts to accelerate progress in reducing hepatitis-related deaths^3,5,41^. High hepatitis-related mortality is not solely a national concern; instead, it calls for broader regional cooperation among countries to effectively combat this public health challenge.

Low mortality rates were concentrated in Europe and the Americas, showing a clustering of low-values over the decades. This suggests that hepatitis mortality was relatively low in developed countries, especially in Europe where abundant health resources are available. Interestingly, the Americas, particularly in Latin America and the Caribbean, also demonstrated notably low numbers and mortality rates. With the exception of Guatemala, countries in the Americas exhibit low hepatitis mortality regardless of their sanitation conditions. Multiple factors contributed to this low hepatitis mortality, including limited transmission through transfusions and unsafe injections, increased immunization coverage, and expanded treatment interventions^9,42,43^. However, the increasing prevalence of unsafe injections in North America, this should raise concerns about the potential risk of widespread hepatitis transmission in region^42^.

Our study investigated the association of socioeconomic factors with hepatitis-related mortality, revealing the important role of health expenditure, per capita income, and universal health coverage. Notably, health expenditure exhibited a considerable correlation with mortality, as reflected by the high *q*-statistics. In 2019, chronic hepatitis, including liver cancer and cirrhosis due to hepatitis, accounted for 91.3% of hepatitis-related deaths^18^. Given its complexity and demand for long-term diagnostic and therapeutic resources, high health expenditure becomes imperative for effective hepatitis treatment^2^. However, achieving high health expenditure in low- and lower-middle-income countries is difficult, explaining the concentration of high deaths and mortality rates in Africa and Asia, where a majority of countries fall under these income groups. Meanwhile, universal health coverage emerges as a crucial factor influencing hepatitis-related mortality globally. Universal health coverage aims to establish comprehensive financial protection for the public to ensure universal access to comprehensive health services^44^. In both developing and developed countries, adequate universal health coverage is often better positioned to protect the public against the impacts of infectious diseases and to avert deaths^44–46^. Moreover, the recent COVID-19 pandemic further highlighted the importance of universal health coverage for hepatitis diagnosis and treatment^47,48^. These results suggest that health expenditure dominates hepatitis-related mortality as a direct determinant of treatment quality^49^, particularly affecting patients in low- and lower-middle-income countries who face greater vulnerability to hepatitis deaths due to inadequate health resources.

The effect of hepatitis B vaccination on reducing hepatitis B deaths has not reached its full potential in many developing countries. The study findings revealed a significant correlation between hepatitis B incidence and hepatitis-related mortality, while the correlation of hepatitis B immunization with hepatitis mortality was not significant. In 2019, WHO estimated that approximately 1.5 million people contracted hepatitis B, accounting for half of all new hepatitis infections, and 70–80% of hepatitis-related deaths were due to hepatitis B^2–4^. This evidence confirmed the significant relationship between hepatitis-related mortality and hepatitis B incidence. However, the correlation result of hepatitis B immunization was not consistent with those of hepatitis B incidence. The global coverage of the three-dose series of hepatitis B virus infant vaccination (HepB3) increased from 29 to 81% between 2000 and 2019^50^. Nevertheless, multiple factors contributed to the lack of a significant association between hepatitis B immunization and hepatitis mortality, such as vaccination inequalities and timing of vaccination. Disparities in hepatitis B vaccination persist both between countries and within a country, with vaccine coverage in one-third of countries worldwide falling below the global average^4^. Developing countries, particularly those in Sub-Saharan Africa, are predominantly affected by these inequalities. Furthermore, developing countries, such as those in South Aisa and Southeast Asia, are relatively late in obtaining sufficient hepatitis B vaccine and achieving adequate vaccination^14^. Notably, the majority of hepatitis B deaths are attribute to chronic hepatitis diseases, including liver cancer and cirrhosis, and the progression from hepatitis infection to these diseases is a lengthy process^1,14^. This indicates that in many developing countries, despite the existing relatively high vaccine coverage, a significant reduction in mortality rates reflecting the impact of the vaccines is yet to be observed^14^. These findings why the hepatitis B vaccine is effective in reducing hepatitis B incidence, yet the correlation with hepatitis mortality does not reach statistical significance. Therefore, it will take a long time to reduce mortality rates solely through vaccination. Treatment interventions remain the primary approach for reducing hepatitis-related deaths in individuals with chronic infection, emphasizing the importance of robust health systems, particularly in developing countries.

In recent years, governments and international organizations have initiated various policies and programs to combat viral hepatitis^3, 5^. However, limited attention has been given to hepatitis-related deaths, especially from a geographical perspective. This study employs geographical statistical methods to reveal significant geographical disparities in hepatitis-related mortality, particularly in underdeveloped countries. Our findings suggest that these disparities mainly originate from the differences in economic development, and that economic-related inequalities are prevalent on a global scale^11,49^. Furthermore, the COVID-19 pandemic has added greater challenges to the existing public health infrastructure worldwide^47^. Equitable distribution of health resources, expanded interventions and increased universal health coverage have become more important than ever. To reduce hepatitis-related deaths, global and regional cooperation in health and economics should be strengthened, and effort to balance the distribution of medical and health resources.

There were several limitations to this study. First, this study selected hepatitis-related socio-economic indicators from the SDGs, which may not comprehensively reflect the impact of various risk factors on hepatitis mortality rates. In future research, GBD factors should be included in correlation analysis to better capture the effects of different risk factors on hepatitis mortality rates. Second, hepatitis-related deaths are difficult to ascertain and are systematically underestimated^51^. Even when data were collected from WHO, these estimated hepatitis-related deaths data may not accurately represent the true number of hepatitis-related deaths, particularly in countries where reliable data sources are lacking, such as Afghanistan, Chad and Yemen. This reduced the accuracy of our spatial stratified heterogeneity analysis results, but our results are still applicable to account for the macroscopic effects of health-related status due to the principle of geographical detector. Third, the choice of map projection in this study may affect the spatial statistics results. In general, equal-area cylindrical projections, such as the Equal Earth map projection, are best suited for spatial statistical analysis on global scales. However, most equal-area cylindrical projections, which work on a global scale, are not suitable for showing the hepatitis-related mortality rates in countries. To give a comprehensive picture of hepatitis-related mortality in each country, we used the Compact Miller projection in this study. The Compact Miller projection is an eclectic cylindrical projection that is also suitable for spatial statistical analysis on global scales. Moreover, the results using the Compact Miller projection are highly similar to those using the Equal Earth map projection.

In conclusion, this study demonstrated the elevated hepatitis-related deaths and decreasing hepatitis mortality with significant spatial–temporal variations across countries in the world. It provides essential information for the strategic planning of health services towards accomplishing the World Health Organization target of hepatitis elimination by 2030. By using the geographical detector, we demonstrated the significant associations of health expenditure and universal health coverage with hepatitis-related deaths. Notably, individuals in less developed countries still face a considerable higher risk of death from viral hepatitis. With hepatitis causing over 1.1 million deaths globally annually and being widespread in developing countries, addressing health inequalities and promoting equitable distribution of health resources emerge as critical factors to potentially mitigate hepatitis-related deaths. The results provided valuable insights for optimizing health resource allocation in hepatitis control and reducing hepatitis deaths. The result of this study provided us with novel insights as how to effectively reduce mortality due to hepatitis at global and regional level. Globally, health inequality should be strengthened to reduce, as health-related policies in SDGs are strive to achieve.

### Supplementary Information

Supplementary Tables.

## Data Availability

Data are available in a public, open access repository. See: https://www.who.int/data/gho.
