# Supplementary material for: Spatial–temporal analysis and spatial drivers of hepatitis-related deaths in 183 countries, 2000–2019
Source: Sci Rep. 2023 Nov 13;13:19845. doi: 10.1038/s41598-023-45672-5 (PMC10645816; doi:10.1038/s41598-023-45672-5)
Supplement: Supplementary file 1 — Supplementary Tables. [file 41598_2023_45672_MOESM1_ESM.pdf]

Spatial-temporal analysis and spatial drivers of hepatitis-related deaths in 183 countries, 2000-2019

Jie Li[1,2], Zejia Xu[1], Hong Zhu[1]*

1 School of Geographical Sciences and Remote Sensing, Guangzhou University, Guangzhou, 510006, China
2 School of Geography and Planning, Ningxia University, Yinchuan, 750021, China

* Corresponding author Email: zhuhong@gzhu.edu.cn

Supplementary Table S1 Number of hepatitis-related deaths and percentage in deaths between 2000 and 2019, by location.

Location	2000	2010	2015	2019	Percentage change from 2000 to 2019
**World**	**1141684**	**1108797**	**1116145**	**1153613**	**1.0%**
**Africa**	**184031**	**204252**	**207216**	**212004**	**15.2%**
Algeria	2045	2208	2345	2616	28.0%
Angola	3573	3684	4015	4009	12.2%
Benin	1541	1638	1527	1550	0.6%
Botswana	355	277	271	268	-24.6%
Burkina Faso	2531	2194	2407	2406	-4.9%
Burundi	1336	1237	1184	1288	-3.6%
Cabo Verde	74	78	77	84	12.9%
Cameroon	3767	3726	3182	3260	-13.5%
Central African Republic	1163	1029	955	865	-25.6%
Chad	1709	2245	2309	2307	35.0%
Comoros	91	99	107	114	25.3%
Congo	771	785	689	673	-12.6%
Côte d'Ivoire	3942	3815	3364	3308	-16.1%
Democratic Republic of the Congo	10697	11903	11812	12205	14.1%
Djibouti	117	150	178	191	63.5%
Egypt	41433	54459	61452	60833	46.8%
Equatorial Guinea	120	129	151	150	25.2%
Eritrea	737	836	758	746	1.2%
Eswatini	168	239	178	165	-1.5%
Ethiopia	16776	17413	18768	19842	18.3%
Gabon	300	287	301	280	-6.7%
Gambia	275	346	380	439	59.9%
Ghana	4212	5165	4876	5048	19.8%
Guinea	1881	2245	2417	2369	26.0%
Guinea-Bissau	317	319	319	310	-2.2%
Kenya	6589	7246	6859	8197	24.4%
Lesotho	365	437	552	493	34.8%
Liberia	706	760	757	776	9.9%
Libya	450	437	480	535	18.8%
Madagascar	2295	2919	2927	3242	41.3%
Malawi	3426	2920	2173	2085	-39.2%
Mali	2242	2122	1944	2042	-8.9%
Mauritania	438	454	486	538	22.8%
Mauritius	149	166	132	128	-14.2%
Morocco	2699	2934	3253	3622	34.2%

Mozambique	3438	3771	3253	3460	0.6%
Namibia	213	226	240	196	-8.1%
Niger	1926	2653	2907	2953	53.3%
Nigeria	22863	25105	23517	23913	4.6%
Rwanda	2784	1697	1708	1845	-33.7%
Sao Tome and Principe	27	29	25	27	-0.1%
Senegal	1668	1773	1796	1917	14.9%
Seychelles	18	18	19	21	15.3%
Sierra Leone	1000	1072	1014	1063	6.3%
Somalia	2688	3133	3098	3221	19.8%
South Africa	6179	6953	6427	6264	1.4%
South Sudan	835	1208	1316	1406	68.5%
Sudan	4675	4108	4195	4230	-9.5%
Togo	1089	1241	1176	1088	-0.1%
Tunisia	814	951	1027	1109	36.2%
Uganda	3995	3628	3339	3678	-7.9%
United Republic of Tanzania	6194	5067	4121	4495	-27.4%
Zambia	3080	2892	2830	2696	-12.5%
Zimbabwe	1254	1825	1620	1435	14.4%
**Central Asia**	**9818**	**10476**	**10176**	**9858**	**0.4%**
Kazakhstan	2824	2950	2836	2565	-9.2%
Kyrgyzstan	941	1128	1053	906	-3.8%
Tajikistan	766	896	929	1035	35.1%
Turkmenistan	822	1011	957	967	17.6%
Uzbekistan	4464	4491	4401	4386	-1.8%
**East Asia**	**402374**	**280629**	**274721**	**289507**	**-28.1%**
China	339617	216325	214387	231439	-31.9%
Democratic People's Republic of Korea	5841	6506	6957	6952	19.0%
Japan	41911	42479	38767	37321	-11.0%
Mongolia	1039	1469	1565	1736	67.2%
Republic of Korea	13966	13850	13045	12060	-13.6%
**Europe**	**88647**	**99357**	**93745**	**90666**	**2.3%**
Albania	453	329	325	363	-19.9%
Austria	797	1013	991	818	2.7%
Belarus	736	1221	873	790	7.3%
Belgium	905	931	984	960	6.1%
Bosnia and Herzegovina	457	518	481	481	5.3%
Bulgaria	1138	948	1006	1002	-12%
Croatia	826	688	594	590	-28.5%
Czechia	1207	1321	1086	1191	-1.4%
Denmark	387	491	439	433	11.8%
Estonia	158	150	143	149	-5.9%
Finland	396	562	575	572	44.6%

France	7437	7863	7956	7879	5.9%
Germany	8549	7635	7666	7410	-13.3%
Greece	1233	1208	1444	1693	37.2%
Hungary	3545	2298	1918	1814	-48.8%
Iceland	6	9	15	15	135.3%
Ireland	172	235	244	259	50.4%
Italy	12984	12759	12983	12635	-2.7%
Latvia	207	241	231	240	15.9%
Lithuania	295	428	374	329	11.4%
Luxembourg	38	41	42	45	20.7%
Malta	14	22	23	25	79.4%
Montenegro	42	49	50	45	6.5%
Netherlands	534	617	717	808	51.3%
North Macedonia	225	241	224	223	-0.6%
Norway	153	198	223	213	39.3%
Poland	4143	3805	3754	4174	0.7%
Portugal	1113	1022	1033	1027	-7.8%
Republic of Moldova	1336	1405	1191	1048	-21.6%
Romania	5511	5255	4468	4721	-14.3%
Russian Federation	15844	24270	22102	19450	22.8%
Serbia	1086	957	864	863	-20.5%
Slovakia	775	706	763	749	-3.3%
Slovenia	417	341	290	296	-28.9%
Spain	5412	5836	5728	5413	0.0%
Sweden	423	517	562	562	32.8%
Switzerland	465	514	602	601	29.3%
Ukraine	6778	9130	6327	6468	-4.6%
United Kingdom	2450	3584	4455	4313	76.0%
**North America**	**45026**	**55970**	**64545**	**67021**	**48.9%**
Antigua and Barbuda	4	3	4	3	-7.4%
Bahamas	15	21	20	24	59.2%
Barbados	15	12	15	16	5.0%
Belize	17	18	22	27	59.4%
Canada	1530	2301	2822	2913	90.3%
Costa Rica	182	222	267	317	74.4%
Cuba	587	850	936	1053	79.5%
Dominican Republic	532	584	921	1048	96.9%
El Salvador	306	386	532	507	65.6%
Grenada	6	5	7	6	-1.6%
Guatemala	1625	2182	2631	2797	72.2%
Haiti	932	1053	1115	1190	27.6%
Honduras	534	681	1002	961	79.8%
Jamaica	68	79	84	90	32.2%

Mexico	11749	12367	12731	13844	17.8%
Nicaragua	294	486	561	659	124.3%
Panama	116	141	200	196	68.6%
Saint Lucia	9	9	10	11	23.0%
Saint Vincent and the Grenadines	3	6	5	6	69.6%
Trinidad and Tobago	70	76	71	68	-3.0%
United States of America	26431	34487	40588	41285	56.2%
**Oceania**	**1866**	**2516**	**3400**	**3595**	**92.6%**
Australia	939	1304	1954	2025	115.7%
Fiji	62	84	89	95	53.5%
Kiribati	23	25	26	28	19.7%
Micronesia	16	16	18	20	26.9%
New Zealand	153	188	257	271	77.0%
Papua New Guinea	522	720	857	949	81.8%
Samoa	19	20	21	22	17.3%
Solomon Islands	68	87	99	104	53.3%
Tonga	28	27	28	27	-4.1%
Vanuatu	37	45	50	54	46.4%
**South America**	**25481**	**28349**	**30004**	**31240**	**22.6%**
Argentina	3760	3214	3710	3662	-2.6%
Bolivia	837	847	977	1108	32.3%
Brazil	13374	15700	16580	17105	27.9%
Chile	1878	2178	1974	2021	7.7%
Colombia	1498	1609	1754	1931	28.9%
Ecuador	690	1012	1006	1074	55.6%
Guyana	79	75	71	76	-3.6%
Paraguay	158	234	243	280	77.2%
Peru	1903	2039	2127	2413	26.8%
Suriname	42	45	45	56	33.3%
Uruguay	197	151	186	185	-5.9%
Venezuela	1064	1244	1330	1327	24.8%
**South Asia**	**229841**	**259615**	**256632**	**258911**	**12.6%**
Bangladesh	19161	16663	14692	16139	-15.8%
Bhutan	100	93	102	112	11.6%
India	166110	194705	191548	190045	14.4%
Maldives	24	26	32	31	28.2%
Nepal	2576	2923	2415	2772	7.6%
Pakistan	38912	43461	46442	48388	24.4%
Sri Lanka	2957	1744	1401	1425	-51.8%
**Southeast Asia**	**126129**	**138944**	**143943**	**156393**	**24.0%**
Brunei Darussalam	23	36	44	52	121.5%
Cambodia	4533	6052	5947	6488	43.1%
Indonesia	57453	58583	60434	67201	17.0%

Lao People's Democratic Republic	1171	1165	1288	1406	20.1%
Malaysia	2822	3732	4221	4873	72.7%
Myanmar	10291	12635	12590	13743	33.5%
Philippines	8457	9402	9274	9749	15.3%
Singapore	510	593	695	778	52.3%
Thailand	13560	17410	19679	21549	58.9%
Timor-Leste	98	114	117	124	27.1%
Viet Nam	27210	29221	29655	30430	11.8%
**Western Asia**	**28472**	**28689**	**31764**	**34419**	**20.9%**
Afghanistan	5487	4932	4838	5093	-7.2%
Armenia	350	555	606	508	45.0%
Azerbaijan	1399	1619	1596	1608	14.9%
Bahrain	71	83	93	127	77.1%
Cyprus	40	43	48	48	20.6%
Georgia	1059	965	1019	957	-9.6%
Iran	4607	4780	6219	6831	48.3%
Iraq	1363	1556	1700	1789	31.3%
Israel	363	347	388	346	-4.8%
Jordan	290	288	348	429	48.0%
Kuwait	80	110	151	222	178.9%
Lebanon	368	498	637	723	96.4%
Oman	209	307	336	382	82.7%
Qatar	58	103	127	163	181.2%
Saudi Arabia	2782	3068	3651	4022	44.6%
Syrian Arab Republic	1016	1167	1149	1253	23.3%
Turkey	6230	5581	6225	6885	10.5%
United Arab Emirates	123	300	338	403	228.6%
Yemen	2576	2384	2295	2631	2.1%

Supplementary Table S2 Hepatitis-related mortality rate and percentage change in mortality rate per 100,000 between 2000 and 2019, by location.

Location	2000	2010	2015	2019	Percentage change from 2000 to 2019
**World**	**23.59**	**19.88**	**17.63**	**16.29**	**-30.9%**
**Africa**	**41.32**	**34.03**	**29.47**	**26.68**	**-35.4%**
Algeria	12.07	8.80	7.60	7.23	-40.1%
Angola	50.31	38.76	35.59	31.14	-38.1%
Benin	43.53	35.23	28.18	25.29	-41.9%
Botswana	39.57	23.89	20.43	17.77	-55.1%
Burkina Faso	51.37	34.35	31.93	27.60	-46.3%
Burundi	50.28	34.48	28.09	26.32	-47.7%
Cabo Verde	29.97	22.99	20.22	20.45	-31.8%
Cameroon	47.28	36.55	27.50	25.07	-47.0%
Central African Republic	61.07	48.80	45.18	39.21	-35.8%
Chad	43.57	41.02	37.16	33.48	-23.2%
Comoros	34.84	29.04	26.82	25.28	-27.5%
Congo	51.38	38.37	30.32	25.77	-49.8%
Côte d'Ivoire	47.51	37.64	29.35	26.06	-45.2%
Democratic Republic of the Congo	47.23	39.43	33.43	30.3	-35.8%
Djibouti	32.69	29.29	28.87	26.84	-17.9%
Egypt	99.6	102.29	102.47	90.42	-9.2%
Equatorial Guinea	35.51	25.89	24.95	22.98	-35.3%
Eritrea	56.21	49.10	40.95	37.66	-33.0%
Eswatini	33.48	39.76	29.01	25.16	-24.9%
Ethiopia	54.49	42.26	39.34	36.46	-33.1%
Gabon	39.18	31.31	27.82	22.96	-41.4%
Gambia	43.57	40.08	37.87	39.02	-10.4%
Ghana	47.99	41.51	33.03	30.12	-37.2%
Guinea	45.70	46.35	44.45	38.95	-14.8%
Guinea-Bissau	54.60	44.43	39.44	34.00	-37.7%
Kenya	50.65	42.56	33.59	33.08	-34.7%
Lesotho	31.36	34.07	38.08	31.80	1.4%
Liberia	48.29	39.88	33.99	30.31	-37.2%
Libya	16.43	12.41	12.01	11.75	-28.5%
Madagascar	32.60	29.63	24.83	23.82	-26.9%
Malawi	63.43	46.35	31.11	26.66	-58.0%
Mali	42.89	32.35	26.20	24.48	-42.9%
Mauritania	35.48	26.90	24.12	22.89	-35.5%
Mauritius	14.04	12.06	8.46	7.51	-46.5%
Morocco	14.60	11.62	11.07	10.71	-26.6%
Mozambique	36.97	32.47	25.04	23.43	-36.6%
Namibia	23.36	18.31	17.68	13.48	-42.3%

Niger	37.92	36.66	33.76	29.86	-21.3%
Nigeria	38.68	35.54	29.11	26.53	-31.4%
Rwanda	69.23	36.16	31.07	29.22	-57.8%
Sao Tome and Principe	40.97	34.20	25.79	25.15	-38.6%
Senegal	36.10	30.03	26.02	24.44	-32.3%
Seychelles	27.12	19.62	18.74	18.80	-30.7%
Sierra Leone	42.11	33.45	28.28	26.20	-37.8%
Somalia	62.15	55.00	48.56	45.31	-27.1%
South Africa	20.85	18.17	14.86	12.96	-37.9%
South Sudan	28.42	26.51	25.26	25.60	-9.9%
Sudan	32.65	23.00	20.28	18.43	-43.6%
Togo	44.86	38.94	31.65	25.88	-42.3%
Tunisia	10.99	9.63	9.11	8.76	-20.3%
Uganda	43.22	30.31	24.13	22.46	-48.0%
United Republic of Tanzania	38.72	26.30	18.87	17.69	-54.3%
Zambia	69.87	52.58	43.94	37.01	-47.0%
Zimbabwe	24.42	31.49	25.57	20.93	-14.3%
**Central Asia**	**24.66**	**23.60**	**20.06**	**17.42**	**-29.4%**
Kazakhstan	20.90	19.19	16.74	13.74	-34.3%
Kyrgyzstan	27.25	28.41	23.58	18.46	-32.3%
Tajikistan	21.02	21.11	19.32	18.57	-11.7%
Turkmenistan	27.19	27.23	22.04	20.02	-26.4%
Uzbekistan	26.94	22.05	18.60	16.33	-39.4%
**East Asia**	**37.54**	**33.36**	**28.81**	**26.69**	**-28.9%**
China	29.37	14.28	12.31	11.95	-59.3%
Democratic People's Republic of Korea	29.26	25.47	23.83	21.85	-25.3%
Japan	18.11	13.49	10.64	9.42	-48.0%
Mongolia	80.58	92.30	80.54	76.76	-4.7%
Republic of Korea	30.36	21.26	16.72	13.49	-55.6%
**Europe**	**8.62**	**7.81**	**6.81**	**6.40**	**-25.8%**
Albania	16.97	9.12	7.76	7.77	-54.2%
Austria	6.44	6.71	5.82	4.56	-29.2%
Belarus	6.06	9.73	6.50	5.62	-7.3%
Belgium	5.35	4.68	4.68	4.24	-20.7%
Bosnia and Herzegovina	9.72	8.95	8.40	7.92	-18.5%
Bulgaria	9.38	7.82	8.31	8.31	-11.4%
Croatia	12.48	9.41	7.68	7.48	-40.0%
Czechia	8.39	8.00	6.15	6.55	-21.9%
Denmark	5.11	5.48	4.32	3.93	-23.0%
Estonia	8.39	7.67	7.15	7.23	-13.8%
Finland	4.88	6.21	5.66	5.34	9.4%
France	8.12	7.00	6.35	5.84	-28.0%
Germany	6.56	4.93	4.58	4.30	-34.5%

Greece	6.43	5.19	5.42	5.56	-13.5%
Hungary	25.71	15.1	11.85	10.91	-57.6%
Iceland	1.72	1.90	2.70	2.56	49.4%
Ireland	3.80	4.10	3.75	3.49	-8.4%
Italy	12.01	9.37	8.48	7.68	-36.1%
Latvia	6.26	7.71	7.70	8.05	28.7%
Lithuania	6.79	9.94	8.80	7.73	13.8%
Luxembourg	6.05	5.18	4.58	4.45	-26.4%
Malta	2.54	2.96	2.80	2.74	7.8%
Montenegro	5.59	5.48	5.27	4.49	-19.7%
Netherlands	2.35	2.20	2.29	2.36	0.5%
North Macedonia	9.77	8.69	7.49	6.96	-28.8%
Norway	2.24	2.45	2.50	2.24	0.2%
Poland	8.66	7.01	6.57	7.23	-16.5%
Portugal	7.40	5.60	5.13	4.78	-35.3%
Republic of Moldova	29.41	27.96	22.31	18.61	-36.7%
Romania	18.72	16.86	13.54	14.19	-24.2%
Russian Federation	8.75	12.91	11.17	9.34	6.7%
Serbia	8.24	6.71	5.77	5.64	-31.6%
Slovakia	12.23	9.46	9.52	8.77	-28.3%
Slovenia	15.12	10.06	7.38	7.00	-53.7%
Spain	7.95	6.83	6.14	5.34	-32.8%
Sweden	2.58	2.93	2.96	2.72	5.7%
Switzerland	4.06	3.73	3.84	3.44	-15.2%
Ukraine	11.07	15.03	10.26	10.55	-4.7%
United Kingdom	2.80	3.51	3.86	3.54	26.7%
**North America**	**9.12**	**8.21**	**8.50**	**8.02**	**-12.0%**
Antigua and Barbuda	5.91	3.81	4.10	2.94	-50.3%
Bahamas	6.59	6.57	5.52	5.82	-11.8%
Barbados	4.04	2.99	3.35	3.47	-14%
Belize	14.06	9.65	9.75	9.97	-29%
Canada	3.71	4.31	4.70	4.46	20.4%
Costa Rica	6.18	5.12	5.08	5.20	-15.9%
Cuba	4.62	5.44	5.39	5.61	21.6%
Dominican Republic	9.93	7.84	10.43	10.43	5.0%
El Salvador	7.54	7.44	9.18	8.01	6.2%
Grenada	6.86	3.94	5.66	4.65	-32.2%
Guatemala	26.49	25.89	26.43	24.4	-7.9%
Haiti	18.60	16.72	15.80	15.12	-18.7%
Honduras	15.95	14.65	17.96	15.01	-5.9%
Jamaica	2.97	2.85	2.80	2.80	-5.7%
Mexico	17.25	13.28	11.76	11.41	-33.8%
Nicaragua	11.35	13.69	13.35	13.58	19.7%

Panama	5.22	4.53	5.29	4.46	-14.6%
Saint Lucia	6.71	5.14	4.68	4.67	-30.4%
Saint Vincent and the Grenadines	3.77	5.15	4.44	4.57	21.1%
Trinidad and Tobago	6.36	5.37	4.40	3.80	-40.3%
United States of America	7.37	7.95	8.51	8.11	9.9%
**Oceania**	**23.45**	**20.72**	**20.58**	**19.68**	**-16.1%**
Australia	3.78	4.05	5.43	5.09	34.5%
Fiji	12.56	12.57	12.83	12.55	-0.1%
Kiribati	45.11	36.67	35.13	33.36	-26%
Micronesia	26.33	23.14	23.39	24.00	-8.8%
New Zealand	3.15	3.04	3.58	3.45	9.5%
Papua New Guinea	16.41	16.41	16.76	16.49	0.5%
Samoa	17.63	15.69	15.6	15.24	-13.5%
Solomon Islands	30.76	28.08	26.91	24.91	-19.0%
Tonga	43.01	38.02	36.67	33.49	-22.1%
Vanuatu	35.74	29.52	29.52	28.18	-21.1%
**South America**	**9.87**	**8.13**	**7.32**	**7.05**	**-28.6%**
Argentina	9.50	6.86	7.36	6.73	-29.1%
Bolivia	15.11	11.36	11.2	11.14	-26.3%
Brazil	10.19	8.71	7.87	7.16	-29.7%
Chile	13.42	11.38	8.86	8.00	-40.4%
Colombia	5.55	4.19	3.79	3.57	-35.6%
Ecuador	8.38	8.67	7.24	6.69	-20.2%
Guyana	15.88	12.75	10.74	10.46	-34.2%
Paraguay	5.08	5.30	4.80	4.91	-3.3%
Peru	11.23	8.86	7.65	7.27	-35.2%
Suriname	12.50	10.45	9.20	10.30	-17.6%
Uruguay	4.68	3.27	3.87	3.60	-23.0%
Venezuela	6.92	5.70	5.20	4.78	-30.9%
**South Asia**	**24.40**	**18.8**	**16.33**	**15.39**	**-36.9%**
Bangladesh	25.64	17.20	12.89	12.64	-50.7%
Bhutan	27.20	19.47	18.46	17.95	-34%
India	21.11	19.34	17.27	15.60	-26.1%
Maldives	16.99	12.80	11.71	10.03	-41.0%
Nepal	17.09	15.67	12.28	12.80	-25.1%
Pakistan	45.44	38.61	35.72	33.31	-26.7%
Sri Lanka	17.34	8.48	6.00	5.40	-68.9%
**Southeast Asia**	**32.07**	**27.88**	**24.86**	**23.90**	**-25.5%**
Brunei Darussalam	16.50	16.84	15.47	14.55	-11.8%
Cambodia	74.68	69.15	58.23	56.28	-24.6%
Indonesia	40.11	33.71	30.29	29.40	-26.7%
Lao People's Democratic Republic	43.42	34.22	31.99	30.76	-29.2%
Malaysia	20.74	18.55	17.08	16.92	-18.4%

Myanmar	31.15	30.53	26.98	26.91	-13.6%
Philippines	20.34	15.35	13.07	12.05	-40.8%
Singapore	13.93	10.35	9.55	8.69	-37.6%
Thailand	23.90	22.13	21.62	21.53	-9.9%
Timor-Leste	20.43	19.10	17.10	16.35	-20.0%
Viet Nam	47.60	36.73	32.08	29.51	-38.0%
**Western Asia**	**19.50**	**15.57**	**14.01**	**13.18**	**-32.4%**
Afghanistan	60.91	40.16	33.76	30.86	-49.3%
Armenia	10.62	15.10	15.62	12.48	17.6%
Azerbaijan	23.75	22.11	18.75	16.60	-30.1%
Bahrain	28.77	16.74	13.47	14.89	-48.3%
Cyprus	3.46	2.90	2.86	2.53	-26.9%
Georgia	18.85	17.25	18.05	16.71	-11.4%
Iran	13.03	9.38	10.03	9.67	-25.8%
Iraq	12.11	11.05	10.09	9.41	-22.3%
Israel	5.47	3.65	3.73	3.03	-44.5%
Jordan	13.32	8.74	7.71	7.92	-40.5%
Kuwait	9.89	9.72	8.30	8.59	-13.1%
Lebanon	14.71	12.50	11.84	11.35	-22.8%
Oman	21.35	21.65	17.43	15.55	-27.1%
Qatar	26.42	22.39	20.25	18.81	-28.8%
Saudi Arabia	33.33	25.18	22.92	21.21	-36.4%
Syrian Arab Republic	14.83	12.02	11.26	11.59	-21.8%
Turkey	13.04	8.81	8.17	7.76	-40.5%
United Arab Emirates	12.81	11.88	11.76	11.21	-12.5%
Yemen	33.83	24.68	20.16	20.15	-40.4%
